# Rapid preparation of high-purity nuclear proteins from a small number of cultured cells for use in electrophoretic mobility shift assays

**DOI:** 10.1186/s12865-014-0062-z

**Published:** 2014-12-20

**Authors:** Yuqian Luo, Takeshi Hara, Yuko Ishido, Aya Yoshihara, Kenzaburo Oda, Masahiko Makino, Norihisa Ishii, Naoki Hiroi, Koichi Suzuki

**Affiliations:** Laboratory of Molecular Diagnostics, Department of Mycobacteriology, Leprosy Research Center, National Institute of Infectious Diseases, 4-2-1 Aoba-cho, Higashimurayama-shi, 189-0002 Tokyo Japan; Department of Mycobacteriology, Leprosy Research Center, National Institute of Infectious Diseases, 4-2-1 Aoba-cho, Higashimurayama-shi, 189-0002 Tokyo Japan; Leprosy Research Center, National Institute of Infectious Diseases, 4-2-1 Aoba-cho, Higashimurayama-shi, 189-0002 Tokyo Japan; Department of Education Planning and Development, Faculty of Medicine, Toho University, Tokyo, 143-8540 Japan

**Keywords:** Nuclear protein, Transcription factor, NF-κB, Electrophoretic mobility shift assay

## Abstract

**Background:**

Highly purified nuclear protein is required when using an electrophoretic mobility shift assay (EMSA) to study transcription factors, *e.g.* nuclear factor-κB (NF-κB), a major transcription factor that regulates both innate and adaptive immune responses following infection. Although many protocols have been developed for nuclear protein extraction, they are not necessarily optimized for use in EMSA, often require a large number of cells and long processing times, and do not always result in complete separation of the nuclear and cytoplasmic fractions.

**Results:**

We have developed a simple, rapid and cost-effective method to prepare highly purified nuclear proteins from a small number of both suspended and adherent cultured cells that yields nuclear proteins comparable to those prepared by a standard large-scale method. The efficiency of the method was demonstrated by using EMSA to show the successful detection, in multilple concurrent samples, of NF-κB activation upon tetradecanoyl phorbol acetate (TPA) stimulation.

**Conclusions:**

This method requires only a small number of cells and no specialized equipment. The steps have been simplified, resulting in a short processing time, which allows researchers to process multiple samples simultaneously and quickly. This method is especially optimized for use in EMSA, and may be useful for other applications that include proteomic analysis.

## Background

Infection and subsequent initiation of the innate immune response result in a rapid secretion of inflammatory cytokines through activation of various transcription factors. Among those transcription factors, nuclear factor-κB (NF-κB), which usually exists as a heterodimer formed between subunits p50 and RelA/p65, plays a central role in both innate and adaptive immune responses [1-3]. In unstimulated cells, NF-κB exists in the cytoplasm as an inactive form sequestered by the inhibitor protein IκB [4]. Upon stimulation by a variety of stimuli, including bacterial lipopolysaccharide (LPS) [5], tumor necrosis factor α (TNF-α) [6], interleukin-1β (IL-1β) [7] and reactive oxygen species (ROS) [8], IκB is degraded by IκB kinase (IKK), thus unmasking nuclear localization signals (NLS) that allow NF-κB to enter the nucleus, where it orchestrates the transcription of specific genes [9].

The electrophoretic mobility shift assay (EMSA), a common affinity electrophoresis technique used to study protein-nucleic acid interactions, is often employed to demonstrate the binding of the active form of nuclear NF-κB to its DNA recognition sequence at several time points following a particular stimulation. The first and possibly the most crucial step in ensuring accurate detection of NF-κB binding and stoichiometry by EMSA is purification of nuclear protein extracts that contain DNA-binding proteins with no contamination by the cytoplasmic fraction.

Many basic protocols for the extraction of whole nuclear protein, and modified protocols to further separate subnuclear proteins (*e.g.* nucleoplasmic proteins, nucleolar proteins, and histone proteins), have been published in the 70 years since subcellular fractionation was introduced [10-22]. Today, a wide range of commercial products, although much more costly, are available for more convenient application of subcellular fractionation, and a number of procedures have been optimized for use in proteomic studies [14,23-25]. Indeed, nuclear protein extraction procedures should be optimized for starting material (cultured cells or tissues), scale (numbers of cells and samples), downstream applications and available time and cost. However, we noted several drawbacks when using previously reported procedures. They were laborious and time-consuming, required large (15 ml) centrifuge tubes, and necessitated a large number of cells. In response, we developed a novel EMSA protocol that allows examination of the binding and stoichiometry of nuclear NF-κB in a small quantity of cultured cells (*e.g.* cells from one well in a 6-well plate).

We describe here a new small-scale method that can yield ready-to-use high-purity nuclear proteins optimized for use in EMSA. It is rapid and cost-effective, allowing the simultaneous and rapid processing of multiple samples in the same batch experiment. The method is highly efficient, as demonstrated by the simultaneous detection of NF-κB activation and binding in multiple samples of THP-1 human monocyte cells and FRTL-5 rat thyroid epithelial cells upon stimulation of tetradecanoyl phorbol acetate (TPA).

## Results and discussion

### New homogenization method for small-scale preparation of nuclear extracts

**Figure 1:**
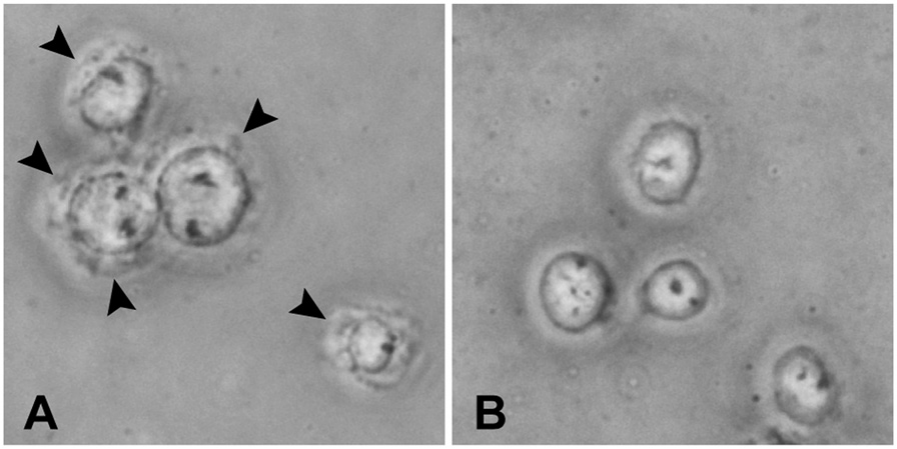
**Efficient release of nuclei from cells using hypo-osmotic buffer and pipetting.** Phase-contrast microscopic image of THP-1 cells in Lysis Buffer before **(A)** and after **(B)** pipetting through a 200-μl pipette tip. Original magnification: ×200. Arrowheads indicate membrane components around the nuclei that were observed before **(A)**, but not after **(B)**, pipetting.

The basic principle underlying subcellular fractionation procedures is that each cellular organelle or component (*e.g.* cytoplasm and nucleus) has a distinct molecular composition, size, shape, density, and solubility. The first step in preparing nuclear proteins is to gently break open, or homogenize, the cells, enabling separation of the cytoplasm and nucleus. Homogenization can be achieved by osmotic shock, mechanical force, sonication, or combinations of these techniques. We modified previously reported methods [15,26] and developed a new homogenization protocol that can be used with a small quantity of cells (5×10^5^ cells). In this modified procedure, collected cells are resuspended in a hypo-osmotic lysis buffer, while 2% Tween-40 (a non-denaturing nonionic detergent) solubilizes and disrupts cytoplasmic membranes. However, hypo-osmotic lysis buffer alone is often insufficient to ensure full release of nuclei from cells, which, in our experience, is the most important step for avoiding contamination by cytosolic proteins. As shown in Figure 1A, human monocytic leukemia THP-1 cells suspended in the hypo-osmotic lysis buffer still have membrane components (arrowheads) around the nuclei, indicating a need for mechanical force.

Mechanical force to rupture cells is most often achieved using the glass Dounce homogenizer [15,22,24,27-30]; however, such specialized equipment is not suitable for a small-scale method. During preliminary experiments, we found that pipetting cells in hypo-osmotic lysis buffer through a conventional 200-μl pipette tip 60–200 times is sufficient to completely release nuclei and yield high-purity nuclear protein in cultured hematopoietic, fibroblasts and epithelial cell lines. Nuclear protein yields may depend on the number of passes: drawing lysate through the pipette tip 100 times gave satisfactory results in all cell lines tested in our preliminary experiments. Microscopic observation is used to determine whether nuclei are completely released from cells and ready for separation from the cytoplasmic fraction (Figure 1B).

### Optimizing separation of nuclear and cytoplasmic fractions

Two types of centrifugation are usually used for fractionation: differential centrifugation and sucrose density gradient centrifugation. It is believed that differential centrifugation yields crude fractions, while purer fractions are obtained from density gradient centrifugation. A sucrose density gradient solution is prepared by overlaying sucrose solutions (at different concentrations) in order of their densities (concentrations) in a centrifuge tube, with the heaviest solution at the bottom. Samples placed on top of the solution will travel down the gradient during centrifugation until reaching a sucrose solution of matching density. Thus, density gradient centrifugation allows further separation of cellular components according to their densities and minimizes cross-contamination. To minimize contamination by the cytoplasmic fraction, we incorporated sucrose density gradient centrifugation into this procedure using an isotonic 0.3-1.5 M sucrose density gradient. The intermediate phase between 0.3 M and 1.5 M sucrose solutions can be retained as cytoplasmic fractions, while pure nuclei extracts are pelleted to the bottom of the tube. Washing nuclei pellets in low-salt wash buffer after sucrose density gradient centrifugation also minimizes contamination from the cytoplasmic fraction.

### High salt extraction is an efficient method for small-scale nuclear protein extraction

In large-scale methods, tip sonication is often applied to extract the nuclear proteins after nuclei extracts are obtained [22,27,30,31]. In a small-scale procedure, high-salt extraction is more practical [23,25]. During this step, nuclei incubated in a high-salt extraction buffer will shrink and nucleic acid-binding proteins, including transcription factors, will be extracted through the nuclear pores and solubilized in the extraction buffer [15]. Following centrifugation, the supernatant (less than 50 μl) contains high-concentration high-purity nuclear proteins. Since this procedure generates a high concentration of nuclear proteins, a small volume of protein can be added directly to the EMSA reaction mixture without a laborious dialysis step to remove salts.

### Validation of the new small-scale nuclear protein extraction method

**Figure 2:**
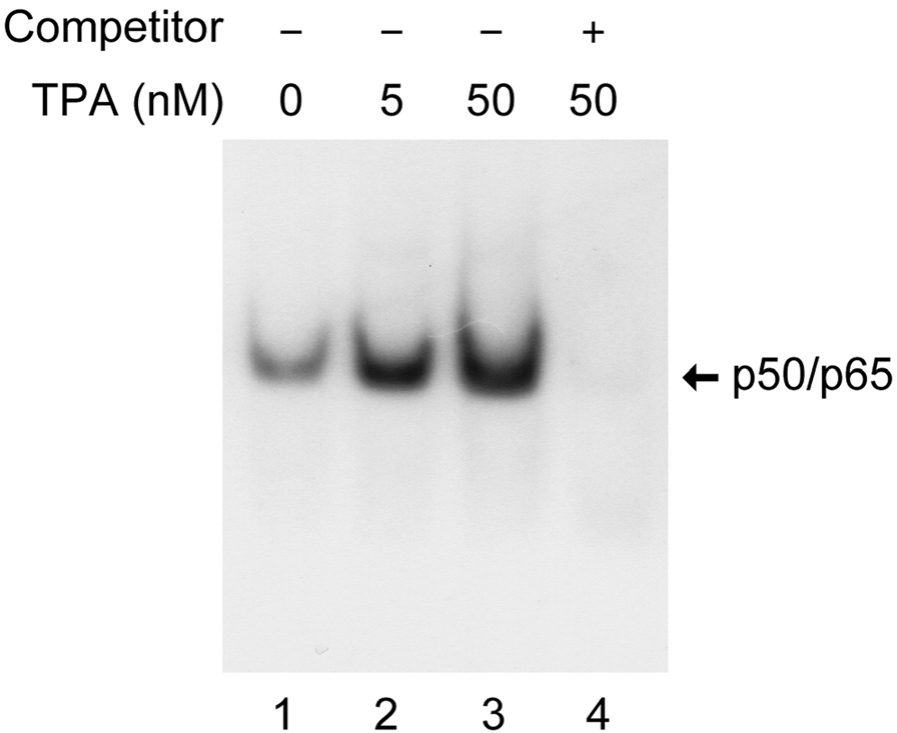
**Nuclear protein extracts contain active NF-κB heterodimers.** Non-adherent monocytic THP-1 cells were stimulated by TPA at the indicated concentrations for 30 minutes. Nuclear proteins were subsequently purified from THP-1 cells and EMSA was performed using an NF-κB-specific DNA probe (lanes 1–3). For specific competition experiments, unlabeled excess (125-fold molar) probe was pre-added to the proteins (lane 4).

Nuclear proteins prepared using this method were used to evaluate the activated form of nuclear NF-κB in THP-1 cells treated with increasing concentrations of TPA, a drug that can induce cell activation, proliferation and cytokine production via stimulation of the NF-κB transcription factor [1,32-36]. THP-1 cells were stimulated with TPA for 30 minutes before nuclear protein was isolated and used in EMSA. As shown in Figure 2, levels of the active form of NF-κB increased with increasing concentrations of TPA (Figure 2 lanes 1–3). When an excess amount of unlabeled probe was added, the band representing the protein-DNA complex was eliminated (Figure 2, lane 4), indicating that the visualized band was indeed formed by NF-κB, not by non-specific protein-DNA binding.

**Figure 3:**
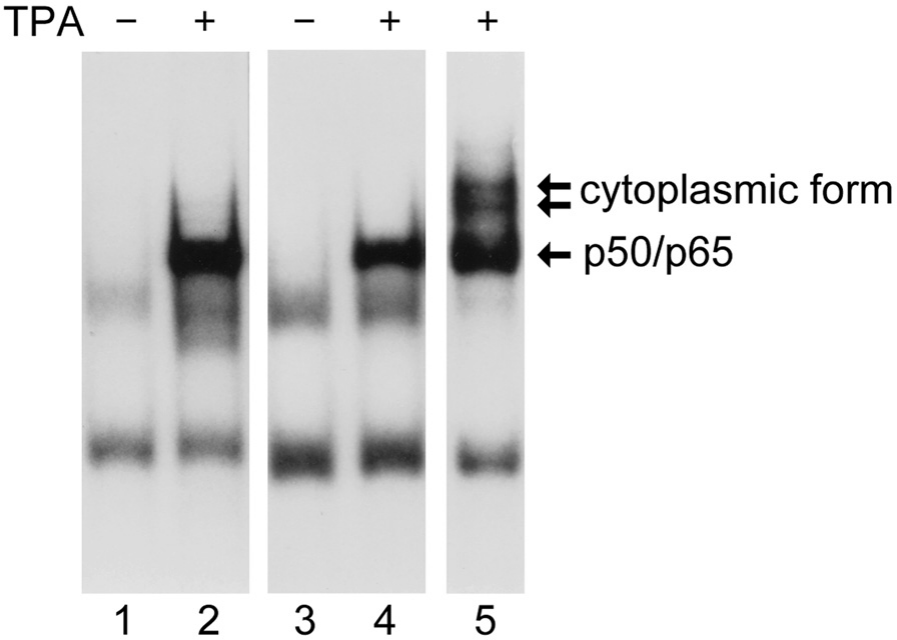
**Nuclear protein extracts are comparable to those prepared by a standard large-scale method.** Adherent rat thyroid FRTL-5 cells were stimulated by 50 nM TPA for 30 minutes. Nuclear proteins were subsequently purified using the new small-scale method (lanes 1 and 2) or an established large-scale method [15] (lanes 3 and 4). Whole cellular protein was also isolated [37] from TPA-treated cells to illustrate the presence of the cytoplasmic form of NF-κB (lane 5). EMSA was performed using an NF-κB-specific DNA probe.

We then tested this method using adherent epithelial cells, from which nuclei release appeared to be more difficult than in hematopoietic THP-1 cells. FRTL-5 cells stimulated by 5 nM TPA for 30 minutes were scraped from a 6-well cell culture plate and pelleted in 1.5-ml microcentrifuge tubes. Nuclear proteins were then purified using our newly developed small-scale method. For comparison, nuclear proteins were also prepared using a standard large-scale method for highly purified nuclear protein extraction that requires at least 1×10^9^ cells and homogenization using a Dounce glass homogenizer [15]. Whole cell proteins containing both active and inactive forms of NF-κB were also prepared using a method described previously [37]. EMSA detection of NF-κB clearly illustrated that the quality of nuclear proteins purified using the present method (Figure 3, lanes 1 and 2) is comparable to that of proteins prepared by the previous large-scale method [15] (Figure 3, lanes 3 and 4) with no contaminating cytoplasmic fractions, which is clearly visible in EMSA using whole cell proteins (Figure 3, lane 5).

Thus, EMSA results clearly demonstrated NF-κB activation after TPA stimulation in two cell types, showing that this simplified small-scale extraction of nuclear proteins is highly effective for downstream EMSA applications.

## Conclusions

We have developed a simplified method of obtaining high-purity nuclear protein for use in EMSA. The method uses significantly fewer cultured cells than current conventional methods (up to 4 logs less). Key procedures are disruption of the plasma membrane by pipetting cells through a conventional 200-μl pipette tip in a hypo-osmic solution containing detergent, separation of nuclei by sucrose density gradient centrifugation, and high-salt extraction of nuclear proteins. Multiple samples can be processed simultaneously as all the procedures are performed in a conventional 1.5-ml microcentrifuge tube. Using this method, nuclear proteins can be prepared in a short time from both suspended and adherent cultured cells, which may be compatible with use in proteomic assays as well.

## Methods

### Cell culture and TPA stimulation

THP-1, a human monocytic leukemia cell line, was obtained from the American Type Culture Collection (ATCC; Manassas, VA) and cultured in RPMI medium supplemented with 10% charcoal-treated fetal bovine serum, 2% nonessential amino acids, and 50 mg/ml penicillin/streptomycin as described [38,39]. Rat thyroid FRTL-5 cells provided by Interthyr Research Foundation (Athens, OH) were maintained in Coon’s modified Ham’s F-12 medium supplemented with 5% bovine serum (Invitrogen, Carlsbad, CA) and a six-hormone mixture as previously described [40,41]. For TPA stimulation, culture medium was replaced, 30 minutes before nuclear protein was extracted, with the same medium containing 5 nM or 50 nM of TPA.

### Nuclear protein extraction

#### Reagents

- Dithiothreitol (DTT) (Sigma cat. No. D0632)
- Dulbecco’s modified phosphate buffered saline (DPBS) (Sigma cat. No. D1283)
- Ethylenediaminetetraacetic acid (EDTA) (Sigma cat. No. EDS)
- Glycerol (Sigma cat. No. G5516)
- HEPES (Sigma cat. No. H3375)
- Leupeptin (Sigma cat. No. L2884)
- Magnesium chloride (MgCl_2_) (Sigma cat. No. M8266)
- Potassium chloride (KCl) (Sigma cat. No. P9333)
- Potassium hydroxide (KOH) (Sigma cat. No. P5958)
- Phenylmethanesulfonyl fluoride (PMSF) (Sigma cat. No. P7626)
- Pepstatin A (Sigma cat. No. P5318)
- Protease inhibitor cocktail tablets (Roche cat. No. 11697498001)
- Sodium chloride (NaCl) (Sigma cat. No. S7653)
- Sucrose (Sigma cat. No. S7903)
- Tween-40 (Sigma cat. No. P1504)

#### Solutions

Low-salt wash buffer: 10 mM HEPES-KOH pH 7.9, 10 mM KCl, 1.5 mM MgCl_2_, 0.1 mM EDTA, 0.5 mM DTT, 0.5 mM PMSF, 2 ng/ml pepstatin A, and 2 ng/ml leupeptin.

Hypo-osmotic lysis buffer: 0.3 M sucrose, 2% (v/v) Tween 40, 10 mM HEPES-KOH pH 7.9, 10 mM KCl, 1.5 mM MgCl_2_, 0.1 mM EDTA, 0.5 mM DTT, 0.5 mM PMSF, 2 ng/ml pepstatin A, and 2 ng/ml leupeptin.

1.5 M sucrose buffer: 1.5 M sucrose, 10 mM HEPES-KOH pH 7.9, 10 mM KCl, 1.5 mM MgCl_2_, 0.1 mM EDTA, 0.5 mM DTT, 0.5 mM PMSF, 2 ng/ml pepstatin A, and 2 ng/ml leupeptin.

High-salt extraction buffer: 20 mM HEPES-KOH pH 7.9, 420 mM NaCl, 1.5 mM MgCl_2_, 0.2 mM EDTA, 25% glycerol, 0.5 mM DTT, 0.5 mM PMSF, 2 ng/ml pepstatin A, and 2 ng/ml leupeptin.

Protease inhibitors including DTT, PMSF, pepstatin A, and leupeptin were added immediately prior to use. On occasion, a protease inhibitor cocktail tablet (Roche) was used instead. Hypo-osmotic lysis buffer and 1.5 M sucrose buffer were prepared by adding sucrose and Tween-40 or sucrose to low-salt wash buffer at the indicated concentrations.

#### Equipment

- Table-top Microcentrifuge (Eppendrof 5415D)
- Inverted routine microscope (Nikon Eclipse TS100) with high-definition digital camera (Nikon DS-Fi1)
- Micropipettes
- Pipette tips
- 1.5-ml microcentrifuge tubes

#### Protocol

In the following procedure, all samples, reagents and tubes were pre-chilled and kept on ice. All centrifugations were performed in a table-top microcentrifuge at 12,000 rpm and 4°C. Typically, 5×10^5^ cells were collected and pelleted by centrifugation for 30 seconds in a 1.5-ml microcentrifuge tube. The supernatants were removed and the cell pellets were resuspended and washed in 1 ml of ice-cold Dulbecco’s modified phosphate buffered saline (DPBS). After another centrifugation, pellet, packed cell volume (pcv) was estimated, and the pellets were resuspended in a volume of hypo-osmotic lysis buffer 5 times the pcv. At this point, samples can be stored at −80°C until needed (thaw in a 37°C water bath prior to use). Cells were homogenized by pipetting 100 times using a micropipette with a 200-μl pipette tip. Enucleated samples were overlaid on 1 ml of 1.5 M sucrose buffer and centrifuged for 10 minutes. Purity of the nuclei and distribution of other cellular components before and after sucrose density gradient centrifugation were checked by examinating a small aliquot of sample under a phase contrast microscope. Supernatants were removed after centrifugation and the nuclear pellets were resuspended in 1 ml of low-salt wash buffer and pelleted again by centrifugation for 30 seconds. After the supernatants were removed, the washed nuclear pellets (retained as cleaner nuclei) were resuspended in 50 μl of high-salt extraction buffer and placed on ice for 20 minutes with occasional vortexing. Following 20 minutes of extraction, the samples were centrifuged for 20 minutes and the supernatants were retained as high-purity nuclear proteins.

### Determination of protein concentration

Protein concentration was determined using *DC* protein assay reagents (BIO-RAD, CA) according to the manufacturer’s instructions [40,41]. Specific absorbance at 750 nm was measured using a VMax Kinetic Microplate Reader (Molecular Devices, Sunnyvale, CA).

### EMSA

EMSA was performed with the DIG Gel Shift Kit, 2nd Generation (Roche, Basel, Switzerland) according to the manufacturer’s instructions. Briefly, a double-stranded DNA probe specific for NF-κB responsive element (5′-AGTTGAGGGGACTTTCCCAGGC-3′) was labeled with digoxigenin-11-ddUTP. Nuclear protein samples (0.2 μg) were mixed with 0.4 ng of labeled DNA probe. For the competition assay, 125-fold molar excess unlabeled DNA probe was premixed with the protein for 20 minutes before labeled DNA probe was added. The mixture was electrophoresed on a 6% (v/v) non-denaturing polyacrylamide gel in 0.5x Tris-boric acid-electrophoresis (TBE) buffer at 4°C. Following electrophoresis, protein was transferred from the gel to a positively charged nylon membrane by electroblotting. Digoxigenin-labeled complexes on the membrane were detected using an alkaline phosphatase-conjugated anti-digoxigenin antibody (1:10,000) and its chemiluminescent substrate disodium 3-(4-methoxyspiro {1,2-dioxetane-3,2′-(5′-chloro) tricyclo [3.3.1.13,7]decan}-4-yl)phenyl phosphate (CSPD), both provided in the kit. Chemiluminescent signals were visualized by exposing the membranes to X-ray film.

All reagents were purchased from Sigma-Aldrich (St. Louis, MO) unless otherwise indicated.
